# Ethical considerations of phone-based interviews from three studies of COVID-19 impact in Bihar, India

**DOI:** 10.1136/bmjgh-2021-005981

**Published:** 2021-08-17

**Authors:** Karima Khalil, Priya Das, Rochana Kammowanee, Deepika Saluja, Priyanjali Mitra, Shamayita Das, Dipwanita Gharai, Dinesh Bhatt, Navneet Kumar, Samuel Franzen

**Affiliations:** 1 Software and Gender, Oxford Policy Management, New Delhi, India; 2 Health System Software and Gender, Oxford Policy Management, New Delhi, India; 3 Sociology, University of Chicago Division of the Social Sciences, Chicago, Illinois, USA; 4 Training and Survey Operations, Oxford Policy Management, New Delhi, India; 5 Training and Survey Operations, Oxford Policy Management, Oxford, UK; 6 Monitoring and Evaluation, Health Portfolio, Oxford Policy Management, Oxford, UK

**Keywords:** COVID-19, health policy, health services research, health systems evaluation

## Abstract

Phone-based interviews present a range of ethical challenges, including how to ensure informed consent and privacy and maintain confidentiality. Our paper presents conceptual and practical ethical considerations taken into account across three telephone studies on the impact of COVID-19 conducted following India’s nationwide lockdown imposed in March 2020. Two studies captured COVID-19 response impact on primary-level Reproductive Maternal Neonatal and Child Health (RMNCH) services and on provider wellness, respectively. The third study focused on how the gendered experience of COVID-19 and the state’s response to control transmission impacted women’s lives, focusing on health services, livelihood, entitlements and social change, by interviewing individual women. The ethical challenges as well as the advantages of digital data collection are presented with recommendations for low-resource settings. Ethical considerations included the above challenges as well as avoiding posing unreasonable time burden on the respondents, framing questions with a gendered lens, considering emotional states given contagion concerns and economic uncertainties, and redressing pandemic-induced distress. Using scripted Hindi was challenging in consent-taking, as was protecting household respondents’ privacy and confidentiality during lockdown. Unanticipated positive ethical implications of using a telephone approach included providing respondents privacy and catharsis, respondents choosing convenient interview times and affording health providers more privacy than institutional inperson interviews. Internalising empathy, respect and appreciative enquiry are key to establishing rapport in the absence of prior relationships. Institutional Review Board (IRB) time limits on call duration need to be flexible to allow for ‘active listening’ and empathetic enquiry in surveys on the impact of COVID-19.

**Summary box**

- Phone-based studies under COVID-19 face numerous ethical challenges around ensuring consent and confidentiality, protecting respondents’ privacy and accessing vulnerable populations.
- Collecting data telephonically can have unanticipated and positive advantages over in- person data collection
- IRB panels need to rapidly develop flexible guidelines that enable remote data collection in a timely manner and that take pandemic conditions and stresses into account.

## Introduction

Public health emergencies necessitate rapid research to inform policymakers and align response efforts with evolving population needs.^1^ Data collection is even more essential during pandemic lockdowns for agile policy response and to generate evidence for postpandemic policies and ensure continuity of time-series analyses.^2^ COVID-19 has led to suspension of routine fieldwork and health surveys due to lockdowns and focus on infection surveillance.^3^ Phone surveys fill the gaps created by disruptions in population-based inperson research, enabling critical documentation of on-ground experiences.^3^

Phone-based data collection presents a unique set of ethical challenges around consent, confidentiality, representativeness, data quality and respondents’ privacy, posing conceptual and practical considerations.^4–6^ Concerns around informed consent for phone-based data collection include communicating consent information clearly and with brevity, considering the time burden on the respondents.^4^ Respondents’ understanding of consent and the challenges faced by researchers in gauging their comprehension in the absence of non-verbal cues are more pronounced.^7^ Confidentiality and data privacy issues in linking contact numbers to personal information are other ethical considerations with phone-based methods.^4^

The absence of visual cues in telephone interviews can affect data quality owing to loss of rich non-verbal data.^8^ This inherent shortcoming compromises data contextualisation and rapport-building with respondents and often requires follow-up with probes.^9 10^ Methodological discussions on telephonic data collection also highlight the lack of indepth responses in order to keep interviews short.^11^ The induced bias of socially desirable responses is another challenge, although not unique to telephone surveys.^12^ However, there is little evidence to suggest that quality or data interpretation is compromised in telephone interviews.^7^

The gender gap in phone ownership as well as the social constraints in their use or access^13^ make ethical considerations regarding data collected by phone in low-income and middle-income countries (LMICs) like India more pronounced. Mobile phone surveys are particularly challenging in LMICs, where digital inequities across income levels and gender impede technology-based data collection.^14^ Capturing the experiences of vulnerable socioeconomically marginalised communities and women in LMICs can be difficult.

Much of the existing literature on the ethics of digital research methods focuses on the global West. Studies that explore improving the efficacy of phone-based data collection in LMICs also emanate from the global West and focus on quantitative methods-oriented technological tools.^15–17^ This paper fills a gap in LMIC-based phone data collection, particularly qualitative phone-based interviews, made more challenging by the crisis. It explores several key ethical conceptual and practical considerations involved in three longitudinal studies of the impact of COVID-19 from the perspectives of healthcare providers and community women in rural Bihar in Eastern India. The paper also presents unanticipated positive implications of the digital shift, with recommendations for future phone-based research in low-resource settings.

## Background

In March 2020 India imposed a nationwide lockdown, which required our data collection to pivot to phone-based studies of the impact of COVID-19. These included two facility provider studies: an ongoing facility ethnography study that shifted from inperson fieldwork at two primary care facilities to phone interviews with providers,^18^ and a longitudinal phone survey of primary-level health providers, the COVID-19 provider study.^19^ There was also a qualitative study of women’s perceptions: Bihar COVID-19: Experiences of Community Women Study.^20^ The latter was facilitated by Project Concern International working with the Bihar Rural Livelihoods Project (BRLP), known locally as JEEViKA, the largest network of women self-help groups (SHGs) in Bihar.

The facility ethnography study was part of a learning exercise to understand contextual factors shaping provider practices at primary-level facilities. The study was conducted as part of the Oxford Policy Management’s ongoing work providing learning and evaluation support to the Bill and Melinda Gates Foundation’s health system strengthening programmes in Bihar. The study initially involved embedding two three-person teams at two facilities, in four sequential rounds of data collection (2019–2020). This study shifted to phone interviews to understand the primary health system response, the impact of COVID-19 on services and the workforce challenges. In chronicling the evolving situation, the team sought to understand the health system’s adaptive capacity and resilience to inform COVID-19 response and recovery.

The COVID-19 provider study, a state-wide phone survey of a sample of primary-level health providers at 95 public facilities across 95 blocks (a block or a community development block is a district subdivision of the rural development department and Panchayati Raj institutes, consisting of a cluster of villages), aimed to understand COVID-19 response preparedness and changes in service delivery, document the impact on essential and Reproductive, Maternal, Neonatal and Child Health (RMNCH) services, and understand provider safety, wellness, challenges and personal resilience, all from the perspectives of providers (doctors and nurses). The study also aimed to document local strategies developed to mitigate the impact of COVID-19 that can benefit others.

The community women study aimed to understand women’s perspectives of the pandemic’s impact at the individual, household and community levels. It explored the gendered experience of COVID-19 by understanding how the pandemic and state response to control transmission impacted women’s lives, focusing on health services, livelihood, entitlements and social change. The purpose was to inform organisations working with the government on the concerns and priorities of women and girls, as the government moves towards the pandemic’s recovery phase. Short qualitative interviews were conducted telephonically with 48 women across two districts and six blocks in rural Bihar using an open-ended interview guide. The respondents included SHG leaders, group members and JEEViKA community mobilisers.

The next section outlines the ethical challenges faced from planning to data analysis and how these were addressed. These included assessing the research burden, acquiring consent to contact respondents by phone, ensuring informed consent to participate, safeguarding privacy, anonymity and confidentiality, minimising time obligations, redressing expressions of pandemic-induced stress or distress, and mitigating data security risks.

## Conceptual and practical ethical challenges and mitigating steps

### Planning

#### Assessing the research burden

An important ethical consideration was assessing the absolute need for the research given COVID-19’s burden on respondents. Bihar’s population experienced lockdown-induced economic hardships, in addition to contagion fears and mass worker migration, with a dynamic, evolving situation changing daily. Health service providers bore the double burden of home stress and heavy facility workload. Research relevance must be questioned under these conditions, avoiding research duplication and targeting similar respondents by consulting research-aggregating platforms like CORE Net (or the COVID-19 Research Network, which is an effort to build a community of practice to foster exchange and collaboration among research organisations gathering information on issues relevant to the COVID-19 pandemic in India) and by collaborating with researchers in geographical settings. This was taken into account by all three studies, with the ultimate decision that each filled relevant and timely knowledge needs.

#### Seeking collaboration

As in all data collection, trust and rapport are essential for quality information exchange. The facility ethnography study’s prior relationship with the respondents placed those interviews within pre-existing rapport, facilitating information-sharing. The two other studies approached the respondents via familiar institutional partners. The COVID-19 provider study attempted to allay respondents’ concerns by having district-level administrators inform them of the impending study and its objectives, with unclear effectiveness. The community women study partially bridged unfamiliarity by approaching women through a partner organisation already working with these women, which contacted respondents and explained the study, who then expected the calls.

### Design

Phone-based research may miss the most vulnerable who lack phone access. In Bihar, the most vulnerable, often rural women, may not have phones or may be unable to independently use a male relative’s phone. The community women study only reached women with their own phones, who were part of the JEEViKA network. Some respondents described other vulnerable women who did not have phones. This obviously has implications for sampling representativeness and relevance of findings if the objective is to reach the society’s most disadvantaged.

#### Consent to initiate contact and safeguarding privacy

Accessing telephone numbers and acquiring consent to be contacted are areas that require navigating with care due to invasion of privacy and potential for coercion. In the facility ethnography study, providers initially shared their contacts and consented to be contacted at the initiation of inperson fieldwork. In the facility-based COVID-19 provider study, accessing phone numbers was more complex. Respondents’ contacts were accessed from the publicly available State Health Society directory and steps were put in place to acquire consent to call them on these numbers by sending an opt-out SMS (short message service) prior to phoning. In the community women study, contacts were accessed through the partner organisation, with women’s verbal consent to be called for a later interview recorded.

For the facility ethnography study, as engagement with respondents was already ongoing, verbal recorded consent was not required. The COVID-19 provider and community women studies both recorded verbal consent to conduct the interviews; the former sent a copy of the consent back to the respondents via SMS to ensure transparency, while the latter also recorded the respondents’ consent to the partner organisation to ensure no coercion was applied.

#### Keeping consent meaningful and short

In designing, it is crucial to consider the length of informed consent statements and word choice.^4^ Simple language is essential while preserving the five core requirements of informed consent, as placing minimum time burden on the respondents is critical. Drafting these was challenging across all three studies due to Institutional Review Board (IRB) standardised text requirements. It was also sometimes challenging for the community women study researchers to gauge respondents’ understanding of consent language or interview questions, necessitating follow-up confirmatory probes and simpler rephrasing.

#### Factoring for a gendered perspective

Ebola and COVID-19 have both revealed pandemics’ gendered effects on women^21^; therefore, designing questions to capture COVID-19’s impact on women requires framing questions with a gendered lens,^22^ and the community women study aimed to capture these. In framing questions on government initiatives, community action or household impact (work burden, economic burden, coping strategies), men’s experiences (as told by women) and women were focused on separately.

### Data collection

#### Catering to respondent availability and minimising the research burden

Researchers across all three studies tried to limit the time burden by conducting interviews at the respondents’ convenience, engaging over two to three calls if the respondents preferred. The COVID-19 provider study removed previously answered background questions from subsequent rounds, replacing these in the interview tool with new questions to stay within time limitations. Avoiding lengthy limitations made comprehensive probing and triangulating issues difficult in all three studies. The COVID-19 provider study limited probing to answering question options, except for open-ended questions. The community women study adapted the interview approach, focusing on issues respondents spoke about at length instead of answering all questions in the tool. All three studies had varied response durations across all rounds, with the same respondents giving varying time in different rounds. Some respondents spoke for 15–20 min, while others—even burdened facility providers—were eager to spend over an hour in several instances.

#### Using translators can impact privacy and confidentiality

Interviewing in local dialects builds trust and creates rapport,^1^ but hiring and training local interviewers was not logistically feasible given the pandemic. The facility ethnography and COVID-19 provider studies faced no language barriers as the respondents were facility-based providers and spoke Hindi. While the community women study faced no issues interviewing community mobilisers or JEEViKA network leaders, language barriers did exclude socioeconomically marginalised respondents speaking local Bhojpuri and Maithili dialects. Translators were not used due to the three-way call challenges and to avoid increasing time burden on the respondents.

#### Trust: rapport-building challenges and the value of facilitating partners

The facility ethnography study’s prior relationship with the respondents placed those interviews on a foundation of pre-existing rapport. The COVID-19 provider study explained consent language in detail, ensuring the respondents fully understood, and scheduled interviews at the respondents’ preferred time. The community women study partially bridged unfamiliarity by approaching women through a partner organisation, which contacted respondents, explained the study and obtained their consent to be called. This notwithstanding, researchers put significant effort in rapport-building, making two call rounds: first obtaining consent to be interviewed and scheduling, followed by the interview call. All studies trained interviewers to be patient and empathetic, to use a conversational tone and to ask after respondents’ well-being. However, to build trust and rapport, consent-seeking often took up to 15–20 min in the latter two studies.

Researchers in all three studies were coached in active listening and maintaining affirmative enquiry, even if respondents gave socially appropriate responses, and to not question or doubt respondents’ credibility in any way. Phone interviews are inherently limited by the absence of visible cues, and spoken language nuances are even more critical.^8^ Interviewers in all three studies were coached to express understanding and sincerity and to be alert to discomfort cues.

#### Maintaining privacy

Awareness of respondents’ contexts is key to ensure privacy and confidentiality. Across our three studies there were two vulnerable groups: facility providers airing facility challenges and community women facing three kinds of vulnerabilities—their responses could reflect negatively on the programme they were part of and on the government’s COVID-19 response or affect their home surroundings. All study teams asked respondents when a good time would be to contact them and called back at another timewhen privacy was easier to ensure.

Sharing sensitive information over the phone can put respondents at risk^21^ even in home settings. Applying a gendered lens means sensitivity to language;^21^ domestic violence, which with COVID-19 has risen globally^23^ as well as in India,^24^ is important to capture. However, guaranteeing respondents are not overheard by family members or violence perpetrators is difficult under lockdown. The community women study avoided direct questions on violence: ‘How have the dynamics in the household changed’? and ‘Has the stress and tension in the household increased and if yes, then why?’ were proxy questions asked instead.

#### Preserving anonymity and representation of all findings

The respondents of the facility ethnography study were open in sharing the problems they faced; this information was carefully anonymised, ensuring that the responses represented the respondents’ real-time concerns without affecting confidentiality. The community women study did not always follow the trend or thematic analysis and ensured that all concerns, even those raised by one woman, were reflected in the findings.

#### Distress redressal

Under the pandemic, researchers must consider contagion fears and economic precarity, and questions should consider respondents’ emotional state. In the facility ethnography study, the interviewers were mindful of structural inequities highlighted by the pandemic and faced by some respondents, taking care that conversations were not confirmatory of their subordinate position. Instead of questions around personal protective equipment (PPE) availability for example, proxy questions asked about changes in equipment supplies and coping with stress. The COVID-19 provider study asked direct questions about the leading causes of stress and how these could best be addressed in the respondents’ views. Some respondents expressed themselves at length; the approach provided them with a much-needed platform to voice worries and concerns. COVID-19 mental health support platforms were shared with respondents in that study and were also available to the respondents of the community women study who expressed the need.

A common misunderstanding encountered by the community women study researchers was the respondents assuming calls were to provide COVID-19 information or assistance. Clearly explaining study purpose, risks and benefits is critical to avoid raising false expectations, and this was done repeatedly. However, some researchers continued to be asked for information and support by community women respondents of comparatively lesser means. Researchers referred these women to the JEEViKA network while being mindful of their concerns, continuing to be empathetic and conducting the interview in a conversational and not in an extractive manner. While the respondents accepted that the researchers could not provide real-time solutions to accessing entitlements or fears about husbands returning to the city for work, some researchers felt an ethical dilemma in not supporting the women in any way.

### Analysis

#### Data quality

The facility ethnography team’s prior embedding yielded rich contextual background information against which to weigh quality of responses, in addition to triangulating information from other study provider interviews and with findings by consortium partners. Other strategies to ensure data quality included review of secondary research and local media reports. Access to phone recordings enables quality checks and better monitoring of interview quality over inperson interviews. One of the takeaways from shifting to phone platforms is reconsidering the framing of data robustness when research is conducted during a time of crisis. The community women study data were also contextualised against gender-focused research by development partners.

#### Minimising data security risks

Safeguards to maintain data security were applied across all studies and included mitigating misuse of contact details as well as securing data while following a ‘privacy by design’ principle across the data responsibility chain.^25^ Interviewers used dedicated phones and all data were treated according to IRB-approved ethical protocols, including strict adherence to anonymity by ensuring no identifying information was used in audio recordings, transcripts or reports. All three studies adhered to confidentiality of respondents’ contact details, with these only shared with the researchers involved, stored in password-protected computers and accessible only to the analysis team.

## Advantages of phone-based data collection

The telephone interviews allowed critical research when inperson methods were impossible, while still maintaining data quality. For health providers for whom institutional settings were a barrier to information-sharing, the privacy afforded by phone interviews offered a platform and a voice to share facility-based challenges they faced both preceding and during the COVID-19 response, which many were keen to express. In the facility-based, inperson phases of the facility ethnography study, ensuring complete privacy was not possible. When the study shifted to phone interviews, the respondents were much more forthcoming about the service delivery challenges they faced, challenges that preceded the pandemic as well as those that resulted from it. Previously hesitant to speak critically in the workplace, these respondents were much more open in the telephone interviews about the challenges posed by the routine, top-down monthly service delivery targets they were expected to reach, for example. They were also open about the scarcity of PPE distribution in the early weeks of COVID-19. Conducting interviews at the respondents’ convenience was another advantage of phone-based data collection. The respondents of the facility ethnography study were often interviewed in the evening, if they specified a preference for this time frame. Another advantage shared by all three studies was splitting the interview over several calls at the respondent’s convenience. This would not have been possible in inperson interviews given the challenging logistics involved in revisiting the remote study sites.

The phone platform offered similar anonymity to women to share their pandemic experiences. The social isolation and the latent loss of agency due to movement limitations meant women were unable to meet and share their concerns with other women. Qualitative phone interviews under these conditions may offer a covert benefit by creating a space for women to voice their concerns and be heard.

Contacting respondents at their convenient time, which is difficult with inperson data collection, is another advantage of phone interviews. Easier monitoring of interview quality is another advantage.

## Conclusion

The gender gap in phone ownership as well as the social constraints in their use or access^13^ make ethical considerations regarding data collected by phone in LMICs like India more pronounced. Mobile phone surveys are particularly challenging in LMICs, where digital inequities across income levels and gender impede technology-based data collection.^14^ Capturing the experiences of vulnerable socioeconomically marginalised communities and women in LMICs can be difficult.

Access to subjects and recruitment in rural areas will always depend on the target population’s characteristics: on the respondent category, on the geography of the locale, on respondents’ literacy and on their degree of autonomy and gender. Where all respondents are familiar with the basic technology, have autonomy and are empowered, recruitment for remote data collection faces a level field and is an effective approach. However, where telephone usage is controlled by societal gender norms, digital inequalities across gender will impede access to subjects, who may be those who are most vulnerable. It will remain challenging to reach constituencies who are not comfortable with using telephones, where only local dialects are spoken or where there is low literacy. In such communities, rapport-building is especially challenging and digital approaches inevitably risk being exclusionary to some degree. Researchers must be aware of the lacunae in who they can reach in such environments and why, and build strategies to ameliorate or minimise these challenges to access wherever possible.

The effectiveness of remote data collection is also dependent on the subject of the study; research about sensitive topics by telephone, such as domestic violence or sexual and reproductive health concerns in adolescents, will remain challenging. Conducting focus group discussions remotely will also remain problematic as the platform does not lend itself to interactive group dynamics and effective information exchange in the same way as face-to face group discussions do.

Under the COVID-19 pandemic, it is important to consider the affective atmosphere before undertaking research and adapting to disruptions in normal routines and the existing uncertainty when shifting to remote fieldwork.^26^ It is also important to carefully consider respondents’ privacy as they may be living in environments of harassment, violence or family surveillance.

Considering vulnerabilities as well as inequalities exacerbated by the pandemic and probing the societal value of research studies conducted with empathy can yield methodologically and ethically sound research based on reflexivity and restraint.^27^

The very short time limits on call duration (sometimes as little as 15 min) imposed by IRB panels and the standardised wordings of consent forms should be more flexible to allow for full explanations of consent, rapport-building and active listening. Unduly burdening respondents with a lengthy interview during a pandemic is a valid IRB concern. However, explaining consent forms adequately, building rapport, establishing trust and conducting an interview can take up to or even more than 45 min. This was the case in the two studies described here that were telephone-based from the inception, where calls routinely ran over the time limits that the IRB panel would have preferred. Explaining complex consent language was very time-consuming in both studies. Interviewers were also flexible regarding call duration, for example with community women respondents who were stressed and who sought reassurance. Similarly, the facility provider study allowed respondents the time they needed if they wished to stay on the line and share their work concerns. Empathetic enquiry in surveys on the impact of COVID-19 requires calls of longer duration than normally allowed given the extreme stress the pandemic is invoking. In the words of a facility ethnography study team member, “To be ethical, we actually need more time, not less.”

IRB bodies need to rapidly develop ethical guidelines enabling remote data collection with amenable processes that may differ from procedures applied in normal situations. IRB bodies also need to adapt to the time-sensitiveness of COVID-19 research enquiries, where situations are rapidly evolving on the ground and where procedural time lags in awarding clearance to go forward result in loss of valuable data capture at critical times during the pandemic.

Going forward, for digital data collection to be maximally effective, it should ideally include a mix of inperson fieldwork, even for a brief period, to establish some degree of rapport before moving to remote interviews. Finally, phone interviewers will always need an ethically based understanding of respondents’ vulnerabilities when conducting research in times of crisis.

## Data Availability

Data are available upon request.
